# Self-Medication and Antimicrobial Resistance: A Survey of Students Studying Healthcare Programmes at a Tertiary Institution in Ghana

**DOI:** 10.3389/fpubh.2021.706290

**Published:** 2021-10-08

**Authors:** Alex K. Owusu-Ofori, Eric Darko, Cynthia A. Danquah, Thomas Agyarko-Poku, Kwame Ohene Buabeng

**Affiliations:** ^1^Department of Clinical Microbiology, School of Medicine and Dentistry, College of Health Sciences, Kwame Nkrumah University of Science and Technology, Kumasi, Ghana; ^2^Department of Pharmacology, Faculty of Pharmacy and Pharmaceutical Sciences, College of Health Sciences, Kwame Nkrumah University of Science and Technology, Kumasi, Ghana; ^3^Department of Pharmacy Practice, Faculty of Pharmacy and Pharmaceutical Sciences, College of Health Sciences, Kwame Nkrumah University of Science and Technology, Kumasi, Ghana

**Keywords:** attitude, knowledge, antibiotics, resistance, self-medication

## Abstract

**Objectives:** Antimicrobial resistance (AMR) is one of the biggest challenges facing mankind. Inappropriate uses of antibiotics including self-medication promote the increase and spread of AMR. Self-medication has not been well-studied among students. This study was undertaken to determine students of healthcare programmes self-medication practices and attitudes in relation to AMR.

**Materials and Methods:** This was a cross-sectional survey that used a pretested self-administered questionnaire to elicit responses from first-year students of healthcare programmes at the Kwame Nkrumah University of Science and Technology, Ghana from January 2018 to August 2019.

**Results:** Two hundred and eighty students were recruited with 264 of them returning the questionnaire, giving a response rate of 94.3%. Majority were female (68.9%) and participants ages ranged from 16 to 34 years with a mean age (SD) of 19.5 (1.88) years. 136 students (56.2%) had previously purchased antibiotics without a prescription and 78.3% expressed satisfaction with the outcome of self-medication. Amoxicillin (78%) was the most frequent antibiotic bought without a prescription. Majority (76.3%) agreed that self-medication can lead to AMR. Majority (77.0%) believed that antibiotic abuse is a problem in Ghana and 94.8% agreed that the introduction of a course in the University on the rational use of antibiotic will help improve student's knowledge and practices.

**Conclusion:** Self-medication is common among participants despite their knowledge that inappropriate use of antibiotic may lead to resistance. Innovative ways including the introduction of new curricula may help to improve knowledge and to curb wrong attitudes and practices related to antibiotic misuse and ultimately to overcome the problem of AMR.

## Introduction

The usage of antimicrobials has saved millions of lives but its pervasive use to treat infections has resulted in antimicrobial resistance (AMR). In recent times, AMR contributes significantly to the increasing burden of healthcare in the various health facilities (1). In hospitals, AMR may account for a yearly mortality of 25,000 (2) and has contributes to a massive economic burden (3) in the world. AMR is now considered as a global health threat and factors such as over-prescription of antibiotics, incomplete administration of antibiotics dosage, overuse of antibiotics in both animals and humans, poor infection control in the health care setting and self-medication have contributed to it (1, 4). Among the identified reasons, self-medication has been a major factor in the spread of AMR in the twenty-first century (5).

Self-medication is the act of acquiring and consuming drugs such as antibiotics without the advice of a medical doctor either for diagnosis, prescription or surveillance of treatment. Wasteful expenditure, increase in morbidities due to adverse events and resistance to antibiotics are some of the consequences of self-medication (5). Most episodes of illnesses in developing countries are treated by self-medication (6) and this has resulted in increases in resistance of pathogens and causes health hazards such as adverse drug reactions, prolonged suffering and drug dependence (7).

The World Health Organization's global action plan or strategy to tackle AMR suggests that the training of all healthcare professionals is very critical (8). Studies have shown that health workers who are supposed to enhance the knowledge and attitude of consumers (9) to reduce the practice of self-medication continued to indulge in such behaviors and practices (5). Since health workers are normally considered as a behavioral model for patients and the society as a whole (10) it is, important to create awareness on AMR throughout their studies (11). A study conducted in India revealed that students of healthcare programmes obtained antibiotics from various unauthorized sources such as over-the-counter with self-prescription and leftover antibiotics from friends (1). About 26 and 29% of health students admitted to self-medication in studies conducted in India (1) and Jordan (12), respectively. Also, a study conducted among medical students in a Southern Iranian university revealed 42% of them to practice self-medication (13). In sub-Sahara Africa, 38% of health students in a University in Northern Nigeria, practiced self-medication (14).

Self-medication has not been well-studied among health students in Ghana and Africa, therefore, there is the need to focus attention on future prescribers and handlers of antibiotics to promote and ensure effective and efficient use of antibiotics in future (1). This study was undertaken to determine students of healthcare programmes knowledge, attitudes, and self-medication practices on antibiotics and antimicrobial resistance.

## Materials and Methods

This study was a cross-sectional, questionnaire-based research conducted among students studying healthcare programmes at Kwame Nkrumah University of Science and Technology (KNUST). The period of this study was from January 2018 to August 2019 and first-year health students reading programs in nursing, midwifery and medical imaging participated. A total of 280 students were approached to participate in this study through convenience sampling. Participants were asked to complete the questionnaire after informed consent has been taken. The questionnaire was piloted and pretested prior to its use.

The questionnaire was made up of five sections and comprised of a total of thirty-one structured questions. The first section was designed to gather information on the demographic characteristics of participants such as their gender, age and others. The second section was designed to elicit responses to determine the self-medication behaviors of participants. The third section comprised of questions related to practices such as the source of procurement of antibiotic, checking of expiry date and instruction, the reason for self-medication, common antibiotics used for self-medication, and others. These set of questions were structured in Yes or No format.

Participant's knowledge on antibiotics use was the fourth section assessed from the self-administered questionnaires in a Yes or No format. The attitude of participants toward the abuse of antibiotics and its effects on them and society as a whole was the final section assessed by the questionnaires. The data of the study were entered into Microsoft Excel 2010 and analyzed with IBM SPSS Statistics 2.0.

## Results

### Socio-Demographic Characteristics of Participants

A total of 264 students responded out of the 280 students approached giving us a response rate of 94.3% (264/280). The majority of the participants were female (68.9%, *n* = 182) and the ages of the participants ranged from 16 to 34 years with a mean age (SD) of 19.5 years (1.88). The age group with the highest number of participants was 16–20 years (61.0%, *n* = 161), with only one person aged more than 30 years (0.4%). Students who have had working experience at the Hospital were 6.8% (*n* = 18) and 71.9% (*n* = 187) of them have family members as Healthcare Workers (Table 1).

**Table 1:** Socio-demographic characteristics of participants.

**Socio-demographic characteristics (*n =* 264)**	**Categories**	**Proportion *n* (%)**
**Gender**		
	Male	82 (31.1)
	Female	182 (68.9)
**Age group**		
	16–20 years	161 (61.0)
	20–24 years	97 (36.7)
	25–30 years	5 (1.9)
	>30 years	1 (0.4)
**Working experience at the hospital**		
	Yes	18 (6.8)
	No	246 (93.2)
**Having a family member as a health worker (*****n =*** **260)**		
	Yes	187 (71.9)
	No	73 (28.1)

### Self-Medication Behaviors Among Participants

Majority (95.5%, *n* = 252) of the health students knew about antibiotics. There were 79.2% (*n* = 209) of students that have received antibiotic prescriptions from doctors. Among participants, 71.6% (*n* = 189) had also received antibiotic prescriptions from other healthcare workers. The purchase of antibiotics without a prescription (self-medication) was seen among 56.2% (*n* = 136) of the students. The checking of instructions for antibiotic use and the expiry date of the antibiotics were always done among 44.5% (*n* = 114) and 56.4% (*n* = 149) of the students, respectively. Participants who conducted laboratory test before the use of antibiotics were 19.5% (*n* = 47). Majority of the self-medicated students (82.4%, *n* = 112) expressed their satisfaction with the result of self-medication and 76.3% (*n* = 193) of the students agreed that self-medication using antibiotics can contribute to the development of antibiotic resistance (Table 2).

**Table 2:** Self- medication behavior among participants.

**Self-medication behaviors**	**Categories**	**Proportion *n* (%)**
Knowledge of antibiotics (*n =* 264)	Yes	252 (95.5)
	No	12 (4.5)
Prescription of antibiotic by a Doctor to	Yes	209 (79.2)
participants (*n =* 264)	No	55 (20.8)
Prescription of antibiotic by other health	Yes	189 (71.6)
workers to participants (*n =* 264)	No	75 (28.4)
Buying antibiotics without prescription	Yes	136 (56.2)
(*n =* 242)	No	106 (43.8)
Checking instruction of use before taking	Yes, always	114 (44.5)
antibiotic (*n =* 256)	Yes, sometimes	127 (49.6)
	Never	15 (5.9)
Checking expiry date of antibiotic before	Yes	149 (56.4)
use (*n =* 259)	Sometimes	80 (30.3)
	No	30 (11.4)
Laboratory test prior to antibiotic use	Yes	47 (19.5)
(*n =* 241)	No	194 (80.5)
Satisfaction with the results of	Yes	112 (82.4)
self-medication (*n =* 136)	No	24 (17.6)
Self-medication can lead to the	True	193 (76.3)
development of antibiotic resistance (*n =* 253)	False	60 (23.7)

**Figure 1:**
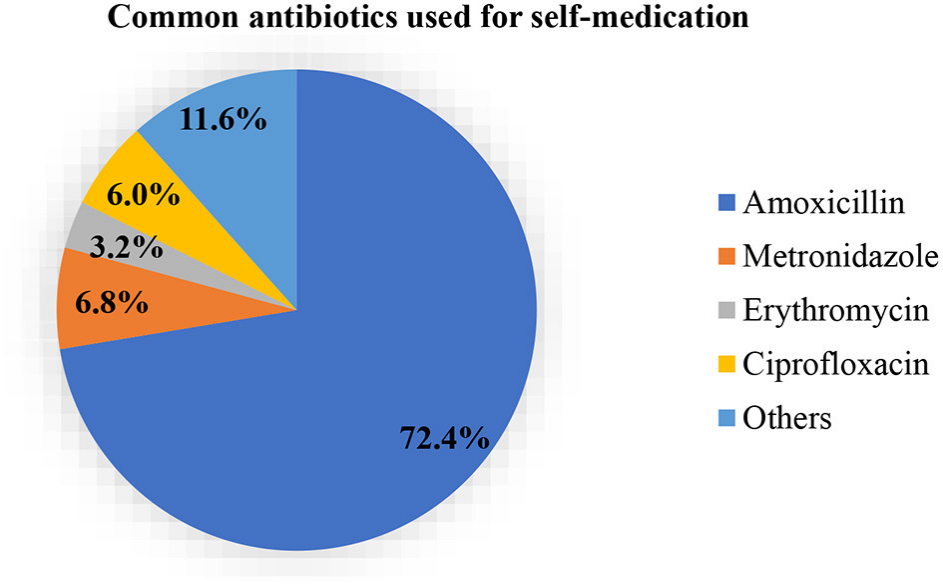
Proportions of common antibiotics used for self-medication.

**Figure 2:**
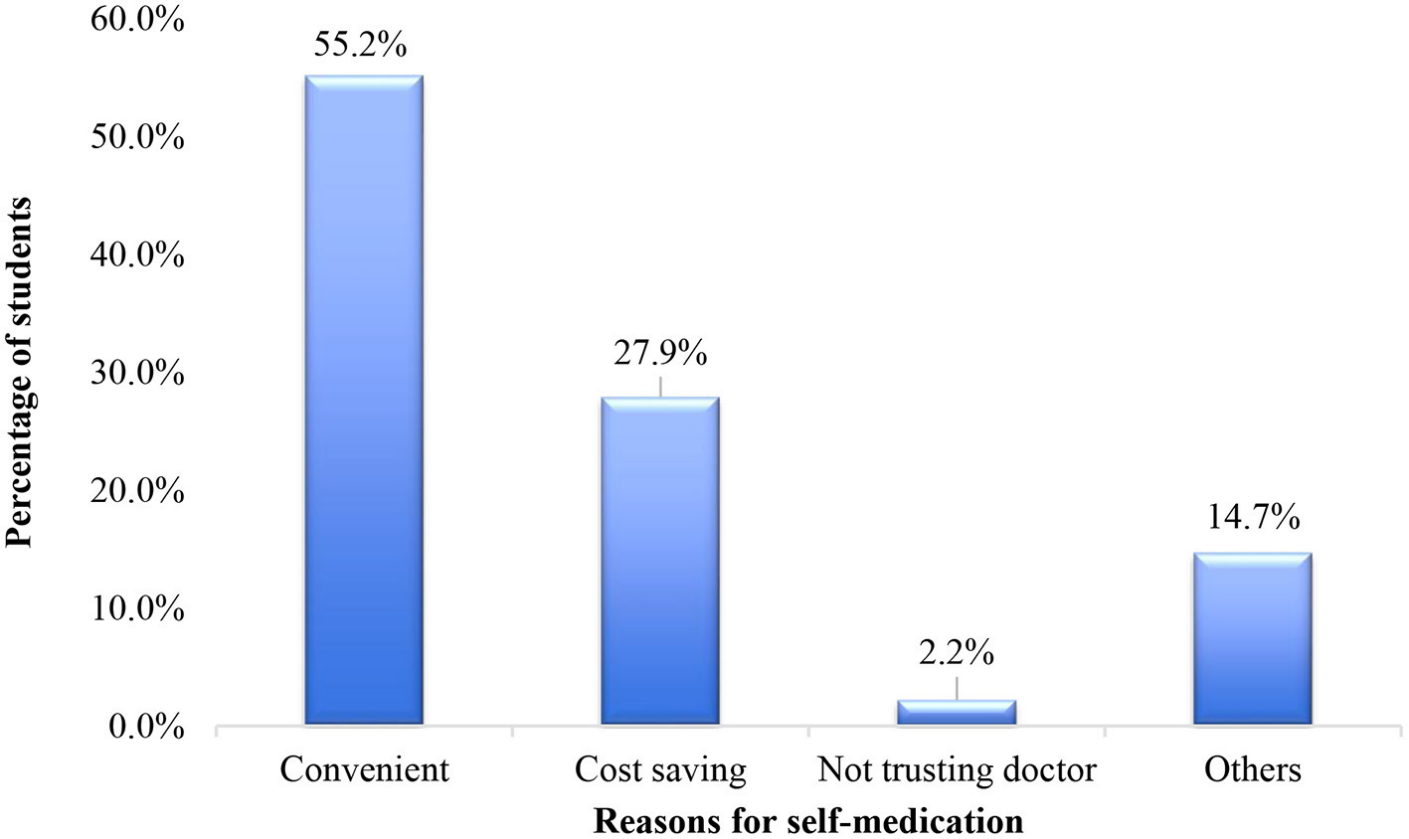
Reasons cited for self-medication among respondents.

**Figure 3:**
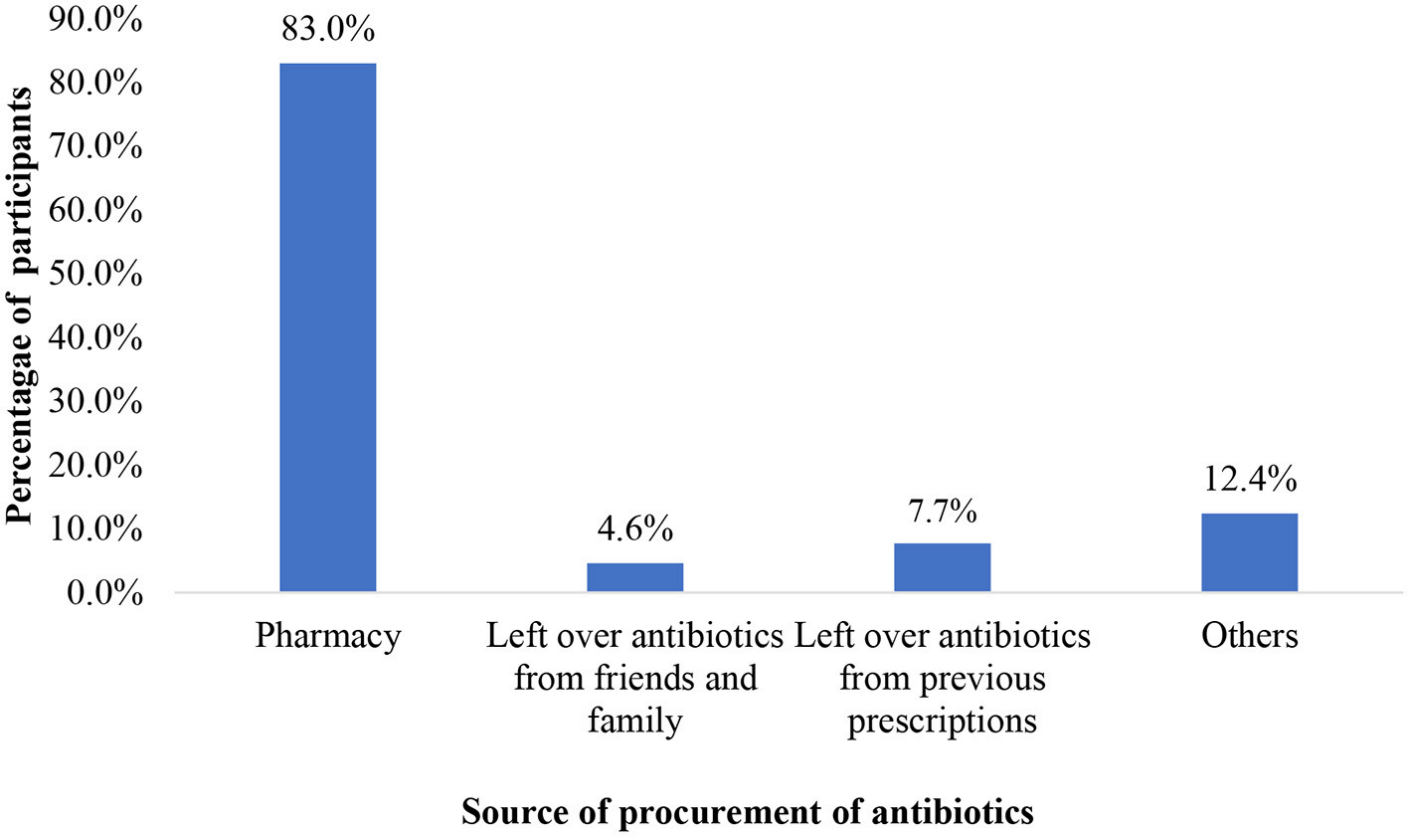
Sources of procurement of antibiotics for self-medication among respondents.

Amoxicillin (72.4%, *n* = 181) was the commonest antibiotic used for self-medication among the participants (Figure 1). Metronidazole (6.8%, *n* = 17), erythromycin (3.2%, *n* = 8) and ciprofloxacin (6.0%, *n* = 15) were the other commonly used antibiotics. Half of the total number of participants cited convenience being the reason for self-medication (55.2%, *n* = 75) and 2.2% (*n* = 3) self-medicated because they did not trust the doctor (Figure 2). Most of the participants (83.0%, *n* = 215) obtained antibiotics for self-medication from the pharmacy (Figure 3).

### Knowledge of Antibiotic Use Among Participants

The use of antibiotics to cure bacterial infections was correctly answered by 94.1% (*n* = 241) of the students. Antibiotics are not used to cure viral infections and this was agreed among 66.9% (*n* = 164) of the students. The knowledge of antibiotic resistance was known among 73.0% (*n* = 187) of the participants and 69.7% (*n* = 177) agreed that frequent use of antibiotic decrease efficacy of antibiotic during treatment (Table 3).

**Table 3:** Knowledge on antibiotic use among participants.

**Parameter (*n =* 264)**	**Categories**	**Proportion *n* (%)**
Knowledge of antibiotic resistance (*n =* 256)	Yes	187 (73.0)
	No	69 (27.0)
Decrease in efficacy of antibiotics	Yes	177 (69.7)
due to the frequent use (*n =* 254)	No	77 (30.3)
Can antibiotics cure bacterial infections?	Yes	241 (94.1)
(*n =* 256)	No	15 (5.9%)
Can antibiotics cure viral infections? (*n =* 245)	Yes	81 (33.1)
	No	164 (66.9)

Again, 92.0% (*n* = 232) and 62.7% (*n* = 158) of the participants who knew about antibiotics (*n* = 252) correctly answered that antibiotics can be used to cure bacterial infections and have no effect on viral infections, respectively.

### The Attitude of Participants Toward the Usage of Antibiotic

There were 90.7% (*n* = 233) participants who had previously misused antibiotics. Among the students, 77.0% (*n* = 194) agreed that antibiotic resistance has become a problem in Ghana. Providing education on antibiotics and the introduction of the rational use of antibiotics at least from the Senior High School (SHS) were agreed by 99.2% (*n* = 251) and 94.8% (*n* = 239), respectively (Table 4).

**Table 4:** Attitude toward antibiotic use among participants.

**Parameter (*n =* 264)**	**Categories**	**Proportion *n* (%)**
Misuse of antibiotics (*n =* 257)	Agree	233 (90.7%)
	Disagree	24 (9.3%)
Inappropriate use of Antibiotic has become a	Agree	194 (77.0%)
problem in Ghana (*n =* 252)	Disagree	58 (23.0%)
Antibiotic resistant affects individual's health	Agree	150 (59.8%)
(*n =* 251)	Disagree	101 (40.2%)
The need to provide information about	Agree	251 (99.2%)
antibiotics (*n =* 253)	Disagree	2 (0.8%)
Introduction of a course on the rational use of	Agree	239 (94.8%)
antibiotics in the University (*n =* 252)	Disagree	13 (5.2%)
Inappropriate use of antibiotics has become the	Agree	190 (75.7%)
main cause of bacterial resistance (*n =* 251)	Disagree	61 (24.3%)

### Practice or Behavior Toward Antibiotic Use Among Participants

Among the participants, 47.6% (*n* = 121) and 63.4% (*n* = 161) used antibiotics to treat common cold and sore throat, respectively. The treatment of conditions such as diarrhea and skin wounds using antibiotics were done by 48.2% (*n* = 122) and 64.7% (*n* = 165), respectively (Table 5).

**Table 5:** Practice or behavior toward antibiotic use among participants.

**Condition (*n =* 264)**	**Categories**	**Proportion *n* (%)**
Common cold (*n =* 254)	Yes	121 (47.6)
	No	133 (52.4)
Sore throat (*n =* 254)	Yes	161 (63.4)
	No	93 (36.6)
Congested nose with headache (*n =* 251)	Yes	126 (50.2)
	No	125 (49.8)
Skin wounds (*n =* 255)	Yes	165 (64.7)
	No	90 (35.3)
Diarrhea (*n =* 253)	Yes	122 (48.2)
	No	131 (51.8)

## Discussion

### Self-Medication Behaviors Among Participants

The majority of the students (95.5%, *n* = 252) have knowledge on what antibiotics are and have used antibiotics to treat their infections. The high knowledge of antibiotics among the students of healthcare programmes in this study was similar to a survey conducted among medical students from School of Medicine in Torino, Italy where more than 90% showed fair knowledge of antibiotics (15). Purchasing of antibiotics should be with the prescription of a doctor and this was seen in the present study where the health students used antibiotics prescribed by doctors (79.2%, *n* = 209) and other healthcare workers (71.6%, *n* = 189). This was similar to 92.8% of students studying healthcare programmes from Southern Indian Teaching Hospital in India who consulted doctors before the administration of antibiotics (16).

Although majority of students have received prescriptions for their antibiotics more than half of the students (56.2%, *n* = 136) have purchased antibiotics on their own without a prescription. The rate was higher in this study than the studies conducted in Karachi, Pakistan, and Torino, Italy, where 35 and 16% of medical students had engaged in self-medication, respectively, (17, 18). The study population of the current research was first-year students of healthcare programmes who were yet to study a course on antibiotics but the study populations in the other works were from various academic year levels. A study conducted by Sawalh in 2008, revealed that college students in Palestine practiced self-medication due to partial knowledge and easy access to antibiotics (19). The finding of this study was higher than 26 and 29% of students of healthcare programmes who admitted to self-medication in studies conducted in India (1) and Jordan (12), respectively. Also, studies conducted among medical students in a Southern Iranian university revealed 42% of them practice self-medication (13) which was lesser than the current study.

Self-medication behavior is one of the ways of misusing antibiotics which eventually lead to the development of resistance in bacteria.

Amoxicillin (72.4%, *n* = 181) was the most commonly used antibiotic for self-medication among the students in the current study and this was similar to a study conducted among Nigerian university students, where amoxicillin (33.0%) recorded the most commonly used antibiotic (20). This could be attributed to the easy availability of the antibiotic on the market. The fight against antibiotic resistance requires multidisciplinary approaches (21). The Country's regulations on the sales of drugs especially antibiotics at the Pharmacy are porous which allows patients or individuals to resort to the Pharmacy as their source of antibiotics without prescription. The current study provided evidence as the Pharmacy (83.0%, *n* = 215) recorded the highest source of procurement of antibiotics for self-medication. Health science students (30.0%) in a university in India were found to have procured antibiotics over the counter without a valid prescription in a study (1). The best practice is to undergo microbiological diagnosis before the administration of antibiotics and this was seen in 19.5% (*n* = 47) of the health students.

One of the reasons for frequent antibiotic abuse is the supposed satisfaction with the results of self-medication. This study revealed that 82.4% (*n* = 112) of the study population were satisfied with the outcome of self-medication. This satisfaction could influence their decision to buy antibiotics on their own without prescription. Strangely, most of the students (*n* = 193, 76.3%) in this study admitted that self-medication contributes to the development of antibiotic resistance but still engaged in self-medication due to reasons such as convenience (55.2%), cost-saving (27.9%), not trusting physician (2.2%) and others (14.7%) for their conduct.

### Knowledge of Antibiotic Use Among Participants

According to a study conducted by Jimah and Ogunseitan in 2020, inadequate knowledge about prudent use of antibiotics among Ghanaians is considered a determinant of increasing risks of antibiotic resistance (22). Regarding participant's knowledge on antibiotic use, 94.1% (*n* = 241) answered correctly that antibiotics can cure bacterial infections. The efficacy of antibiotics normally reduces due to the frequent use of antibiotics and this was agreed by 69.7 (*n* = 177) of the participants. A similarly high percentage (88%) was seen among medical students from the Southern Indian Teaching Hospital, in India, who admitted to the potential reduction in the efficacy of antibiotics due to the frequent use (16).

The wrong notion that antibiotics can cure viral infections was seen among 33.1% (*n* = 81) of the students. From this study, the proportion of student who believed that antibiotics can be used to cure viral infections was higher than a study conducted among professional students of the School of Medicine at the University of Torino where 20.0% believed that antibiotics are appropriate for viral infections (15). The low percentage recorded in Scaioli and his colleagues' study in 2015 could be attributed to the knowledge acquired from a course they took on antibiotics in their first year. A study conducted by Azevedo and his colleagues in 2009 revealed twice the percentage of students (60.0%) in the current study that believed antibiotics can cure viral infections (23). A similar percentage of participants (30.2%) who knew about antibiotics but still use antibiotics to treat viral infection was also seen among students of healthcare programmes (32.0%) in India in a study who believe that antibiotics are effective against viral infections (1). Incorrect and high usage of antibiotics can contribute to high antibiotic resistance in bacteria (23).

### The Attitude of Participants Toward the Usage of Antibiotic

There were diverse attitudes toward the usage of antibiotics among students of healthcare programmes in the current study. Attitudes which result in the inappropriate use of antibiotics eventually lead to the emergence of resistance against the antibiotic. There was a marked level of awareness on antibiotic abuse among health students (90.7%, *n* = 233) in this study. A study conducted in the Democratic Republic of Congo in 2013 revealed that 87% of the medical students and doctors think that antibiotics are overused in their country (24). Among the participants of this study, 77.0% (*n* = 194) believed that the development of resistance in bacteria has become a problem in Ghana and had caused harm to the health of individuals in a community. Similar findings were seen among medical students (88.7%) in India where they admitted antibiotic resistance has caused a great challenge to medical care in their country (16).

The introduction or integration of courses on the knowledge and rational usage of antibiotics into the educational system to curb the misuse of drugs especially antibiotics were agreed among 94.8% (*n* = 239) of the students. This education will eventually shape an individual's attitudes toward antibiotic usage leading to the reduction of antibiotic misuse in future (25). The establishment of a course on the rational use of antibiotics at the University or SHS will help reduce the level of antibiotic abuse in the society (16). Additionally, the education of patients on the need and proper use of antibiotics must be taught among health students.

Despite the high response rate (94.3%) among participants, there were some limitations to the conduction of this study. Firstly, a self-administered questionnaire was used instead of the face-to-face interview which is traditionally considered as the gold standard method of survey administration (26). To give a broad overview of students of healthcare programmes perception on the knowledge, attitude and practice of the use of antibiotics, students from other tertiary institutions can be surveyed in future studies.

## Conclusion

Innovative ways including the introduction of new curricula will help to improve knowledge and to curb wrong attitudes and practices related to antibiotic misuse and ultimately to overcome the problem of antimicrobial resistance.

## Data Availability Statement

The original contributions presented in the study are included in the article/Supplementary Material, further inquiries can be directed to the corresponding authors.

## Ethics Statement

The studies involving human participants were reviewed and approved by Committee on Human Research, Publication and Ethics (CHRPE) at Kwame Nkrumah University of Science and Technology, Ghana. Written informed consent to participate in this study was provided by the participants' legal guardian/next of kin.

## Author Contributions

AO-O: conceptualized and designed the study, supervised, reviewed, and approved the manuscript. ED: obtained, analyzed and interpreted the data, and drafted the original manuscript. CD, TA-P, and KB: reviewed and approved the manuscript. All authors contributed to the article and approved the submitted version.

## Conflict of Interest

The authors declare that the research was conducted in the absence of any commercial or financial relationships that could be construed as a potential conflict of interest.

## Publisher's Note

All claims expressed in this article are solely those of the authors and do not necessarily represent those of their affiliated organizations, or those of the publisher, the editors and the reviewers. Any product that may be evaluated in this article, or claim that may be made by its manufacturer, is not guaranteed or endorsed by the publisher.
