# Supplementary material for: Self-Medication and Antimicrobial Resistance: A Survey of Students Studying Healthcare Programmes at a Tertiary Institution in Ghana
Source: Front Public Health. 2021 Oct 8;9:706290. doi: 10.3389/fpubh.2021.706290 (PMC8534052; doi:10.3389/fpubh.2021.706290)
Supplement: Supplementary file 1 [file Data_Sheet_1.PDF]

KWAME NKRUMAH UNIVERSITY OF SCIENCE AND TECHNOLOGY
COLLEGE OF HEALTH SCIENCES
DEPARTMENT OF CLINICAL MICROBIOLOGY
PILOT STUDY
ASSESSMENT OF HEALTH STUDENTS ON THE USE OF ANTIBIOTICS
QUESTIONNAIRES

Instructions: please tick where applicable and provide details where necessary

Demographic information about the student

1. Gender : [Male] [Female]　　　2. Age:[……]yrs
3. Have you ever worked in a hospital?　　a. Yes　　b. No
4. Do you a family member working as a health worker?　a. Yes　　b. No

Self-medicationBehaviors

1. Do you know about antibiotics?　　a. Yes　　b. No
2. Has a doctor prescribed antibiotics for you before ?　　a. Yes　　b. No
3. Has any healthworker prescribed antibiotics for you before?　　a. Yes　　b. No
4. Have you bought antibiotics on your own without a prescription?　a. Yes b. No
5. What common antibiotic do you take as self-medication?
   a. Amoxicillin　c. erythromycin
   b. Metronidazole/Flagyl　　d. ciprofloxacin
   other (specify)……………………………………………………………

6. Source of procurement of antibiotics
   a. Commonly pharmacy counter
   b. Left over antibiotic from friends and family
   c. Left over antibiotics from previous prescriptions
   d. Other (specify) ……………………………………………………………
7. Do you check the instruction of use before taken antibiotic?
   a. Yes, always　　b. Yes, sometimes　　c. Never　　d. Not applicable

8. Do you check the expiry date of the antibiotic before use?
   a. Yes　　b. No　　c. Sometimes　　d. Not applicable
9. Did you have a lab test prior to antibiotic use
   a. Yes　　b. No　　c. Not applicable
10. Were you satisfied with the results of self-medication using antibiotics?
    a. Yes　　b. No
11. Self-medication using antibiotic can contribute to development of antibiotic resistance
    a. True　　b. false
12. If you have self medicated, what was your reason?
    a. It is convenient　　b. Cost saving　　c. Not trusting doctor
    d. Others (specify)……………………………………………………………

Knowledge on antibiotic use

1. Can antibiotic cure bacterial infections?
   a. Yes b. No
2. Can antibiotic cure viral infections?
   a. Yes b. No
3. Have you heard of antibiotic resistance?
   a. Yes b. No
4. Do you think the frequent use of antibiotic will decrease efficacy of treatment when using the antibiotic again?
   a. Yes b. No

Attitude towards antibiotic use

1. There is abuse of antibiotics
   a. Agree b. disagree
2. Antibiotic resistance has become a problem in Ghana
   a. Agree b. Disagree
3. Abuse of antibiotics has become the main cause leading to bacterial resistant
   a. Agree b. Disagree
4. Antibiotics resistant affects you and your family's health
   a. Agree b. Disagree
5. Is it necessary to provide more information education about antibiotics?
   a. Agree b. Disagree
6. Do we need to establish course on rational use of antibiotics at university or SHS?
   a. Agree b. Disagree

Practice or behavior towards antibiotic use

Do you use antibiotics when having the following?

1. Common cold
   a. Yes b. No
2. Sore throat
   a. Yes b. No
3. Congested nose with headache
   a. Yes b. No
4. Skin wounds
   a. Yes b. No
5. Diarrhea
   a. Yes b. No

Thank you for completing this questionnaire!
